# Evaluation of sinonasal-related quality of life of 49 patients undergoing endoscopic skull base surgery

**DOI:** 10.1016/j.bjorl.2023.101337

**Published:** 2023-10-03

**Authors:** Yijie Zhong, Zeyi Deng, Hailing Chen, Qianhui Qiu

**Affiliations:** a Department of Otorhinolaryngology – Head and Neck Surgery, Guangdong Provincial People's Hospital, Southern Medical University, Guangzhou, China; b Department of Otorhinolaryngology, Central People's Hospital of Zhangjiang, Guangdong, China; c Department of Otorhinolaryngology – Head and Neck Surgery, Guangdong Second Provincial General Hospital, Guangzhou, China; d Department of Otorhinolaryngology, Panyu District Hexian Memorial Hospital, Guangzhou, China

**Keywords:** Endoscopic skull base surgery, Quality of life, Nasal symptoms, Empty nose syndrome, Sleep disorders

## Abstract

**Objective:**

This study aimed to evaluate the sinonasal-related Quality of Life (QoL) in patients undergoing endoscopic skull base surgery.

**Methods:**

A retrospective study was performed, including patients with benign and malignant tumors at a single institution. Each patient completed the 22-Item Sino-Nasal Outcome Test (SNOT-22) and the Empty Nose Syndrome 6 Item Questionnaires (ENS6Q) to assess their perceived QoL at least 2-months after treatment.

**Results:**

Forty-nine patients were enrolled in this study. The average score was 25.1 (Stander Deviation [SD] 14.99) for SNOT-22 and 6.51 (SD = 5.58) for ENS6Q. Analysis of the overall results for the SNOT-22 showed that olfactory damage was the most serious syndrome. The most frequently reported high-severity sub-domains in SNOT-22 were nasal symptoms and sleep symptoms. Nasal crusting was the most severe item in ENS6Q according to the report. Nine patients (18.4%) had a score higher than 10.5 which indicates the high risk of Empty Nose Syndrome (ENS). SNOT-22 score was related to the history of radiotherapy (*p* <  0.05), while the ENS6Q score was not.

**Conclusions:**

The possibility of patients suffering from ENS after nasal endoscopic skull base surgery is at a low level, although the nasal cavity structure is damaged to varying degrees. Meanwhile, patients undergoing endoscopic skull base surgery were likely to suffer nasal problems and sleep disorders. Patients who had received radiotherapy have a worse QoL than those without a history of radiotherapy.

**Level of evidence:**

Level 3.

## Highlights

• Only a small part of patients suffers from ENS after endoscopic skull base surgery.
• Patients undergoing ESBS were likely to suffer nasal problems and sleep disorders.
• Patients who had received radiotherapy have a worse QoL.

## Introduction

Endoscopic Sinus Surgery (ESS) appeared in Austria in the 1970s and was initially only used for the treatment of conventional nasal diseases such as chronic sinusitis, deviation of nasal septum, and nasal polyps. In recent years, with the development of improved endoscopic techniques and technological advancements, endoscopic tumor resection has emerged as the standard for surgical treatment of sinonasal and skull base tumors in appropriately selected cases.^1^ The obvious advantages of this approach are quicker recovery time, better wound healing, increased illumination, visualization of the operative field, and the ability to look around anatomical corners.^2^ As the use of Endoscopic Skull Base Surgery (ESBS) becomes more commonplace for a variety of lesions, there is an increasing push to understand the impact of Quality of Life (QoL) following endoscopic surgery.^3^ Sinonasal Outcome Test-22 (SNOT-22) is widely used as a patient-reported sinonasal QoL instrument in patients undergoing ESBS.^2^, ^4^, ^5^, ^6^, ^7^, ^8^

Empty Nose Syndrome (ENS) is an extremely rare and iatrogenic disease caused by overremoval of functional turbinates with wide nasal cavity and abnormal nasal congestion. The diagnosis of ENS is mainly based on the patient’s symptoms, signs and previous surgical history.^9^, ^10^ However, in recent years, the Empty Nose Syndrome 6 item Questionnaires (ENS6Q) and cotton test have provided new methods for the diagnosis of ENS.^11^ Up to now, few literatures have evaluated the ENS symptoms of patients after endoscopic skull base surgery. The objective of the present study was mainly to evaluate the sinonasal QoL and the ENS symptom of patients receiving endoscopic skull base surgery.

## Methods

### Participants and data collection

This cross-sectional study was approved by the Medical Ethics Committee of Zhujiang Hospital of Southern Medical University. After signing the informed consent, forty-nine patients who underwent endoscopic skull base surgery were routinely asked to complete the 22-item Sinonasal Outcome Test (SNOT-22)^12^ and Empty Nose Syndrome 6 item questionnaire (ENS6Q).^13^

SNOT-22 has been widely used to assess the sinonasal QoL outcomes after endoscopic endonasal skull base surgery.^6^ It has been verified that the scale can be divided into four sub-domains reflecting nasal, otologic/facial pain, sleep, and emotional symptoms.^14^ Nasal symptoms include SNOT-22 items 1–6, 21 and 22. Otologic/facial pain symptoms include SNOT-22 items 7‒10. Sleep symptoms include SNOT-22 items 11‒18. Emotional function includes SNOT-22 items 19 and 20. The Empty Nose Syndrome 6-item Questionnaire (ENS6Q), which includes six nasal symptoms (dryness, sense of diminished nasal airflow, suffocation, nose feels too open, nasal crusting, and nasal burning), was selected as a tool to evaluate the postoperative ENS symptoms.^13^ Based on the subjective feelings of participants, each item of the above scales is divided into six levels which include “no problem”, “very mild”, “mild”, “moderate”, “severe”, and “extremely severe”, with a score of 0–5, respectively.

Patients selected for inclusion were adults over 18-years of age who had undergone endoscopic skull base lesion resection at least 2-months ago. And all included patients had lesions involving sphenoid sinus. Bilateral sphenoidotomies should be performed to remove the floor of the sphenoid sinus, anterior wall, sphenoid rostrum, and intersphenoidal septum. Patients with small lesions who did not open the sphenoid sinus during surgery, patients with a history of endoscopic nasal surgery, and patients who could not complete the questionnaire due to illiteracy were excluded from this study.

Endoscopic surgery methods: The nasal approach of the affected side was usually used. The middle turbinate and posterior ethmoid were removed under the endoscope, and the anterior wall of the sphenoid sinus was exposed. Then, part or all of the inferior turbinate and the inner wall of the maxillary sinus were removed, in order to expose the inner wall of the posterior external wall of the maxillary sinus. The anterior floor of the sphenoid sinus was removed with an electric drill and part of the posterior septum was removed to enlarge the surgical field and facilitate operation. Finally, according to the size of the patient's lesion, the invasive site was resected, except for the regional tissue of important nerves and blood vessels.

All patients filled out the questionnaire by themselves. For individuals unable to finish the questionnaire due to personal reasons, the tester dictated each item to the patient and filled the questionnaire out based on the answers. After the questionnaire completed, we immediately collected the form and made sure that no item was missed.

### Statistical analysis

Statistical analysis was performed using SPSS version 24.0 (IBM corporation, Armonk, NY, USA). To understand the factors that influenced the postoperative scores of patients, a multiple linear regression analysis was performed with age, sex, diagnosis, and history of radiotherapy as independent variables. To evaluate the impact of radiotherapy history on symptom evaluation, we divided 49 participants into groups according to the radiotherapy history. This study involved the comparison of the means of three independent samples that did not follow a normal distribution. The non-parametric Kruskal-Wallis H test was used to compare the SNOT-22 and ENS6Q scale score results. If a difference is found between the groups, the mean rank's post hoc multiple comparison Kruskal-Wallis H test will be performed. The difference was statistically significant when *p* <  0.05.

## Results

A total of 49 patients were eligible for inclusion in this study, 35 (71.4%) of whom were male and 14 (28.6%) were female, with a mean age of 46.3-years (range, 19–50 years). Among 49 patients who underwent surgery, 42 patients underwent intraoperative mucosal flap reconstruction. The nasoseptal flap, the turbinate flap, the artificial skin, and the muscular and fascia tissue were used in 21, 14, 5 and 2 patients, respectively, for the repair of cerebrospinal fluid leakage. The characteristics of patients included in this study are summarized in Table 1. According to the result of multiple linear regression model, only radiotherapy history was correlated with SNOT-22 score (*p* =  0.034). Therefore, we divided the patients into 3 groups according to their radiotherapy history (Table 2). The multiple linear regression model showed that no significant correlations were found between total score of SNOT-22 and the characteristics of patients, including “age”, “gender” and “diagnosis”. However, the SNOT-22 score was significantly correlated with the patient's “radiation therapy history” (*p* =  0.005) (Table 3).

**Table 1:** Characteristic of study participants.

	Study Participants (*n* = 49)
Gender	71.4% (35)
Female	28.6% (14)
Male	46.3 (11.2)
Age, mean in years, (SD)	
Diagnosis	12.3% (6)
Benign	65.3% (32)
Malignant	22.4% (11)
Osteoradionecrosis	
Mucosal flaps	42.9% (21)
Nasoseptal flap	28.6% (14)
Turbinate flap	8.1% (4)
Artificial skin	4.1% (2)
Muscular and fascia tissue	16.3% (8)
No	42.9% (21)
Radiotherapy history	
Never	4.7% (17)
Once	53.1% (26)
Twice (Re-radiotherapy)	12.2% (6)

**Table 2:** Comparison of SNOT-22 and ENS6Q scores of postoperative patients in different radiotherapy groups (`c ± s).

	No radiotherapy (*n* = 17)	Once radiotherapy (*n* = 26)	Re-radiotherapy (*n* = 6)	Total (*n* = 49)	*p*
**SNOT-22 total score**^a^	18.29 ± 11.71	25.27 ± 14.38	43.67 ± 10.56	25.10 ± 14.99	0.002
**Nasal symptoms**	9.18 ± 6.14	10.54 ± 6.50	10.33 ± 4.03	10.04 ± 6.06	0.667
1. Need to “blow” your nose	1.71 ± 1.36	1.85 ± 1.67	2.00 ± 1.41	1.82 ± 1.51	0.887
2. Sneezes	0.82 ± 0.88	0.96 ± 0.92	0.83 ± 1.17	0.90 ± 0.92	0.801
3. Cough	0.41 ± 0.94	0.92 ± 1.23	1.33 ± 1.21	0.80 ± 1.15	0.080
4. “Running” nose	1.12 ± 1.50	0.70 ± 1.09	1.17 ± 0.98	0.90 ± 1.23	0.357
5. Nasal secretion going to your throat	0.65 ± 1.22	1.12 ± 1.48	1.17 ± 1.33	0.96 ± 1.37	0.467
6. Thick secretion from your nose	1.35 ± 1.58	1.08 ± 1.47	0.83 ± 1.60	1.14 ± 1.50	0.661
21. Difficulty to feel “smells” or “tastes”	1.94 ± 1.82	2.73 ± 1.64	2.17 ± 1.33	2.39 ± 1.68	0.312
22. Stuffed nose	1.18 ± 1.38	1.19 ± 1.27	0.83 ± 1.33	1.14 ± 1.29	0.750
**Otologic/facial pain symptoms**^a^	2.24 ± 2.31	3.50 ± 2.70	7.17 ± 3.97	3.51 ± 3.08	0.013
7. A feeling of full or stuffed ear	0.88 ± 1.41	1.31 ± 1.46	1.50 ± 1.64	1.18 ± 1.45	0.456
08. Dizziness or vertigo	0.71 ± 0.69	0.81 ± 1.17	1.00 ± 1.26	0.80 ± 1.02	0.915
9. Ear ach^a^	0.24 ± 0.66	0.65 ± 0.85	2.50 ± 1.87	0.73 ± 1.17	0.002
10. Facial pain or pressure	0.41 ± 0.87	0.73 ± 1.22	2.17 ± 2.04	0.80 ± 1.32	0.068
**Sleep symptoms**^a^	6.29 ± 5.89	9.31 ± 7.53	22.33 ± 8.45	9.86 ± 8.52	0.002
11. Difficulty to sleep^a^	0.88 ± 1.11	1.04 ± 1.31	4.17 ± 1.17	1.37 ± 1.60	0.001
12. Wake up in the middle of the night^a^	0.65 ± 0.93	1.58 ± 1.30	4.33 ± 0.82	1.59 ± 1.58	<0.001
13. Lack of good night of sleep^a^	0.88 ± 0.99	1.46 ± 1.27	4.17 ± 0.98	1.59 ± 1.51	<0.001
14. Wake up tired	0.88 ± 1.22	1.27 ± 1.31	2.00 ± 1.90	1.22 ± 1.37	0.328
15. Fatigued or tired during the day	1.18 ± 1.33	1.15 ± 1.22	2.17 ± 1.33	1.29 ± 1.29	0.224
16. Low performance in doing daily activities	0.35 ± 0.79	0.73 ± 1.28	1.50 ± 2.35	0.69 ± 1.33	0.619
17. Low concentration to do daily activities^a^	0.53 ± 0.72	1.15 ± 1.29	2.17 ± 1.17	1.06 ± 1.20	0.021
18. Frustrated, restless, or irritated	0.94 ± 1.48	0.92 ± 1.26	1.83 ± 1.60	1.04 ± 1.38	0.348
**Emotional function**^a^	0.59 ± 0.80	2.00 ± 2.56	3.50 ± 2.07	1.69 ± 2.23	0.047
19. Sadness	0.47 ± 0.72	0.92 ± 1.35	2.00 ± 1.55	0.90 ± 1.26	0.104
20. A feeling of shame^a^	0.12 ± 0.33	1.08 ± 1.35	1.50 ± 0.84	0.80 ± 1.15	0.005
**ENS6Q total score**	6.47 ± 6.16	6.27 ± 5.10	7.67 ± 6.71	6.51 ± 5.58	0.908
1. Dryness	2.18 ± 1.67	1.38 ± 1.33	2.00 ± 1.10	1.73 ± 1.45	0.223
2. Sense of diminished nasal airflow	0.65 ± 1.11	1.11 ± 1.37	1.33 ± 1.21	0.98 ± 1.27	0.359
3. Suffocation	0.59 ± 1.18	0.58 ± 1.10	1.00 ± 1.67	0.63 ± 1.18	0.883
4. Nose feels too open	1.00 ± 1.41	0.88 ± 1.21	0.83 ± 1.33	0.92 ± 1.27	0.933
5. Nasal crusting	1.29 ± 1.40	1.92 ± 1.49	1.67 ± 1.37	1.67 ± 1.45	0.346
6. Nasal burning	0.76 ± 1.35	0.38 ± 0.85	0.83 ± 1.33	0.57 ± 1.10	0.471

a Statistically significant results.

**Table 3:** Multiple linear regression model results: SNOT-22 and ENS6Q Total Score.

Patient characteristics	SNOT-22		ENS6Q	
	*t*	*p*	*t*	*p*
Age	0.753	0.456	0.776	0.442
Gender	0.536	0.595	1.028	0.310
Diagnosis	0.148	0.883	1.120	0.269
Mucosal flaps	−0.823	0.415	0.151	0.881
Radiotherapy history	2.991	0.005^a^	−0.247	0.806

SNOT-22, Sinonasal Outcome Test 22; ENS6Q, Empty Nose 6 Item Questionnaires.

a Statistically significant results.

All patients were grouped with “radiation therapy history”. The overall average SNOT-22 score was 25.10 ± 14.99 for all of the participants, and the average score of patients with a history of radiotherapy was significantly higher than that without radiotherapy history (*p* =  0.002) (Table 2). The median scores of the patients with different radiotherapy history were 16, 22.5, and 41.5 respectively. Compared with the patients with a history of radiotherapy, those without radiotherapy had significantly lower scores for “ear ache”, “difficulty to sleep”, “wake up in the middle of the night”, “lack of good night of sleep”, “low concentration to do daily activities”, and “a feeling of shame” (*p* = 0.002, *p* =  0.001, *p* <  0.001, *p* <  0.001, *p* = 0.021 and *p* =  0.005, respectively) (Table 2). In the items with significant differences, a pairwise comparison between different groups was performed (Table 4). In the sleep symptoms related items (“difficulty to sleep”, “wake up in the middle of the night”, “lack of good night of sleep”), the scores of re-radiotherapy group were higher than those of non-radiotherapy group and once radiotherapy group, and the difference is statistically significant (*p* =  0.001, *p* =  0.001, *p* <  0.001, *p* = 0.006, *p* <  0.001 and *p* =  0.004, respectively). The scores of “low concentration to do daily activities” were statistically different only between the non-radiotherapy group and the re-radiotherapy group (*p* =  0.018). Among all 22 items, the average score of “difficulty to feel smells or tastes” was the highest with a score of 2.39 ± 1.68, followed by the item “need to blow your nose” with a score of 1.82 ± 1.51. According to different sub-domains, sleep symptoms (“difficulty to sleep”, “wake up in the middle of the night”, “lack of good night of sleep”, “wake up tired”, and “fatigued or tired during the day”) were generally severe, with the average scores of all subjects being 1.37 ± 1.60, 1.59 ± 1.58, 1.59 ± 1.51, 1.22 ± 1.37 and 1.29 ± 1.29, indicating that the patients have different degrees of sleep problems after endoscopic skull base surgery.

**Table 4:** *p*-value of items with differences between the groups after pairwise comparison.

	A + B	A + C	B + C
Ear ache	0.270	0.001	0.038
Difficulty to sleep	1.000	0.001	0.001
Wake up in the middle of the night	0.099	<0.001	0.006
Lack of good night of sleep	0.539	<0.001	0.004
Low concentration to do daily activities	0.393	0.018	0.196
A feeling of shame	0.032	0.011	0.596

A, No radiotherapy; B, Once radiotherapy; C, Re-radiotherapy.

The comparison between groups has been performed Bonferroni correction to adjust the significance value.

In order to further understand the differences in the scores of the four sub-domains of SNOT-22, we also analyzed the scores of the four sub-domains in different radiotherapy groups (Table 2). The results showed that in sub-domains reflecting otologic/facial pain symptoms, sleep symptoms, and emotional function, the scores of patients with a history of re-radiotherapy were significantly higher than those of patients without a history of radiotherapy. The difference was statistically significant (otologic/facial pain symptoms *p* =  0.012, sleep symptoms *p* =  0.02, emotional function *p* =  0.045).

In the ENS6Q scale, the scores of all the participants ranged from 0 to 26, with an average score of 6.51 ± 5.58. There were 9 patients (18.4%) with a score higher than 10.5. The average score of nasal scab (1.80 ± 1.47) is the highest one among the 6 items, which is consistent with the situation we observed under the nasal endoscopy. All of the patients have different degrees of nasal scab in the operation area, mainly in the nasal septum and nasopharynx. The multiple linear regression model showed that no significant correlations were found between total score of ENS6Q and the characteristics of patients, including “age”, “gender”, “diagnosis” and “radiotherapy history” (Table 3). Moreover, no significant difference was found in the scores of ENS6Q items between participants with a history of radiotherapy and that without a history of radiotherapy (Table 2).

## Discussion

By the late 1980s, ESS had gained popularity; endoscopic vision has not only revolutionized the way sinonasal pathology is treated, but has also helped in establishing a better understanding of the anatomy of the paranasal sinuses and beyond.^1^ Nowadays, endoscopic surgery is not limited to the nasal cavity and sinuses, but gradually expands to the more difficult anterior, middle, and posterior skull base areas, which has become “enlarged endoscopic sinus surgery”.^15^ Compared with traditional open approach surgery, nasal endoscopic surgery can directly enter the surgical area through the nasal cavity, which has the advantages of clearer surgical field, more visible operation, and less trauma. Studies have shown that the complications and mortality of nasal endoscopic surgery are lower than those of craniofacial combined anterior skull base surgery.^7^, ^16^ The QoL of patients undergoing endoscopic sinus surgery has long attracted the attention of otolaryngologists. Harrow et al. conducted a 3-year follow-up study of 94 patients underwent nasal endoscopic skull base tumor resection and found that the QoL of patients after surgery was improved, especially in the scores of nasal symptoms and sleep symptoms.^7^ Glicksman et al. evaluated the postoperative QoL in 145 patients who underwent sinus and skull base tumor resection. Although the QoL of the postoperative patients continued to improve within 2-years, the SNOT-22 score of the patients remained high level after 2-years (26.5 and 12.9 for the patients with malignancies and benign tumors, respectively), which showed that the patients will still have different degrees of physical and psychological symptoms for a long time after operation.^8^

In the present study, of 49 patients who underwent endoscopic skull base surgery, 39 (80%) underwent turbinectomy, including 5 bilateral turbinectomy cases and 34 unilateral turbinectomy cases. At the same time, the lesion area must be removed. Almost all of the participants had middle and inferior turbinates defects with different degrees on postoperative imaging examination. The massive loss of turbinate tissue is the pathophysiological basis of ENS.^17^, ^18^ Due to the lack of functional turbinates in the nasal cavity, patients usually suffer paradoxical nasal obstruction despite an objectively patent nasal airway.^19^, ^20^ Velasquez et al. verified that ENS6Q can distinguish patients suspected of ENS, and established a diagnostic scoring standard with a score higher than 10.5.^13^ In the ENS6Q score of this study, only 9 patients (18.4%) had a score higher than 10.5, indicating that only a few patients had a tendency to suffer from ENS after endoscopic skull base surgery although the turbinate and other normal tissues of the nasal cavity were partially lost. Tan et al. and Law et al. also found that resection of middle turbinate does not lead to an increased risk of ENS, especially in the first year.^21^, ^22^ Therefore, we speculate that the cause of ENS is largely related to the psychological factors of patients. Lee et al. also found that anxiety and depression are common psychological disorders in ENS patients.^23^

Radiotherapy is the first choice for the treatment of a variety of head and neck malignant tumor, but it can cause varying degrees of damage to the surrounding tissues. Zhou et al. found that radiotherapy can destroy the nasal mucosal barrier function of patients with nasopharyngeal carcinoma and increase the risk of upper respiratory tract infection in patients.^24^ However, among the 49 participants in this study, there was no significant difference in the nasal symptom scores on the SNOT-22 and ENS6Q scales between patients with a history of radiotherapy and those without a history of radiotherapy. It showed that although radiotherapy had damaged the nasal cavity tissue to a certain extent, for patients who had undergone endoscopic skull base surgery, the history of radiotherapy had no obvious effect on their nasal symptoms. It is worth noting that in the SNOT-22 scores of all patients, the sleep symptom score was at a high level, indicating that postoperative patients were generally troubled by sleep problems. Meanwhile, the sleep symptom scores of patients with a history of re-radiotherapy were significantly higher than those of patients without a history of radiotherapy, suggesting that radiotherapy had a long-term effect on patients' sleep quality.

Radiotherapy, especially re-radiotherapy, also has an adverse effect on emotional symptoms. We reported the scores of the emotional function sub-domain of SNOT-22 and found that patients with radiotherapy had higher emotional-related scores, especially in patients receiving re-radiotherapy. The emotional and cognitive functions of patients after radiotherapy for head and neck malignant tumors will be affected for a long time, which has been mentioned in other QoL-related studies.^25^, ^26^, ^27^ McDowell et al. studied the neurocognitive and neurobehavioral impairment in long-term nasopharyngeal cancer survivors treated with Intensity-Modulated Radiotherapy (IMRT), and found that NPC treated with IMRT had moderate to high rates of neurocognitive impairment and clinically significant apathy, disinhibition, and executive dysfunction. These symptoms were more pronounced in NPC patients who received >75 Gy of temporal lobe radiation. They also found that frontal lobe dysfunction has a strong correlation with lower QoL and higher scores for anxiety and depression.^28^ Therefore, the damage to the brain parenchyma caused by radiotherapy may be a related factor that affects the patient's emotional and cognitive function. However, the relationship between emotional and cognitive function and radiation dose and area still needs to be further elucidated by follow-up research. This also suggests that we should take into account long-term emotional and cognitive dysfunction when IMRT is performed on patients.

The item “difficulty to feel smells or tastes” had the highest score among all measurement items in this study, indicating that change of olfactory is common in postoperative patients. There are two main reasons for postoperative olfactory disorder, including damage to olfactory-related structures and obstructive problems preventing airflow into the olfactory epithelium. Intraoperative damage to the olfactory mucosa and removal of the middle turbinate may be one of the causes of long-term change of olfactory in patients after surgery, because olfactory epithelium also locates on the surface of the turbinate for some people.^29^ Another important reason for olfactory damage is that the common nasal passage is blocked by crusts and secretions in the nasal cavity after surgery, making it difficult for airflow to enter the olfactory area, but the obstructive cause can be significantly improved by long-term nasal irrigation after surgery. Although the history of radiotherapy in this study did not significantly affect the “difficulty to feel smells or tastes” score, the mucosa of the olfactory area is often located on the edge of the radiation field of radiotherapy, and olfactory disorders after radiotherapy have been reported in some literature.^30^, ^31^ Therefore, we suspect that the degree of damage to the sense of smell caused by radiotherapy may be masked by other factors such as the damage of surgery to the sense of smell.

## Conclusion

This study used SNOT-22 and ENS6Q to evaluate the QoL of patients undergoing endoscopic skull base surgery. Postoperative SNOT-22 scores were higher in patients with a history of radiotherapy, especially in the sub-domain of sleep symptoms. This psychological domain of the SNOT-22 appeared to be an area of dim-shined QoL among all patients. Treatment of psychological symptoms may represent an underutilized means of improving postoperative QoL in patients with a history of radiotherapy. Similarly, in terms of nasal symptoms, patients with a history of radiotherapy had more severe olfactory disorders. However, although endoscopic skull base surgery destroys a large amount of normal tissues in the nasal cavity, the ENS symptoms of patients were not that serious.

## Data availability statement

The data that support the findings of this study are available from the corresponding author upon reasonable request.

## Conflicts of interest

The authors declare no conflicts of interest.
